# Coma of unknown origin in the emergency department: implementation of an in-house management routine

**DOI:** 10.1186/s13049-016-0250-3

**Published:** 2016-04-27

**Authors:** Mischa Braun, Wolf Ulrich Schmidt, Martin Möckel, Michael Römer, Christoph J. Ploner, Tobias Lindner

**Affiliations:** Department of Neurology, Charité-Universitätsmedizin Berlin, Campus Virchow-Klinikum, Augustenburger Platz 1, 13353 Berlin, Germany; Center for Stroke Research, Charité-Universitätsmedizin Berlin, Berlin, Germany; Department of Emergency Medicine, Charité-Universitätsmedizin Berlin, Campus Virchow-Klinikum, Berlin, Germany; Department of Anaesthesiology and Intensive Care Medicine, Charité-Universitätsmedizin Berlin, Campus Virchow-Klinikum, Berlin, Germany

**Keywords:** Non traumatic coma, Diagnostic algorithm, Workflow, Brain diseases, Outcome, Neurological emergencies

## Abstract

**Background:**

Coma of unknown origin is an emergency caused by a variety of possibly life-threatening pathologies. Although lethality is high, there are currently no generally accepted management guidelines.

**Methods:**

We implemented a new interdisciplinary standard operating procedure (SOP) for patients presenting with non-traumatic coma of unknown origin. It includes a new in-house triage process, a new alert call, a new composition of the clinical response team and a new management algorithm (altogether termed “coma alarm”). It is triggered by two simple criteria to be checked with out-of-hospital emergency response teams before the patient arrives. A neurologist in collaboration with an internal specialist leads the in-hospital team. Collaboration with anaesthesiology, trauma surgery and neurosurgery is organised along structured pathways that include standardised laboratory tests and imaging. Patients were prospectively enrolled. We calculated response times as well as sensitivity and false positive rates, thus proportions of over- and undertriaged patients, as quality measures for the implementation in the SOP.

**Results:**

During 24 months after implementation, we identified 325 eligible patients. Sensitivity was 60 % initially (months 1–4), then fluctuated between 84 and 94 % (months 5–24). Overtriage never exceeded 15 % and undertriage could be kept low at a maximum of 11 % after a learning period. We achieved a median door-to-CT time of 20 minutes. 85 % of patients needed subsequent ICU treatment, 40 % of which required specialised neuro-ICUs.

**Discussion:**

Our results indicate that our new simple in-house triage criteria may be sufficient to identify eligible patients before arrival. We aimed at ensuring the fastest possible proceedings given high portions of underlying time-sensitive neurological and medical pathologies while using all available resources as purposefully as possible.

**Conclusions:**

Our SOP may provide an appropriate tool for efficient management of patients with non-traumatic coma. Our results justify the assignment of the initial diagnostic workup to neurologists and internal specialists in collaboration with anaesthesiologists.

## Background

Disorders of consciousness including coma in non-traumatic patients can be caused by a wide variety of pathologies affecting the central nervous system (CNS) including potentially life-threatening and highly time-sensitive medical, neurological or neurosurgical emergencies [1]. They are a frequent challenge in emergency medicine: 5–9 % of all patients in emergency departments present with acute non-traumatic disorders of consciousness and up to 2 % are reported to be comatose on admission [2–4]. Moreover, they are associated with a very high in-hospital mortalitiy of 25–48 % in western populations [5, 6]. Underlying pathologies have been classified into primary or focal damage to the CNS and secondary affection of the CNS resulting in a diffuse brain dysfunction, such as in metabolic derangement or intoxication [7]. The reported prevalence of structural vs. metabolic coma varies from 28 to 64 and 37 to 75 %, respectively [8]. Presumptive diagnoses need to be confirmed or excluded promptly in order to treat the patient as quickly as possible or to move on to the next diagnostic step. In many cases, a good outcome critically depends on early treatment (“time is brain”), e.g. in meningo-encephalitis [9] or basilar artery occlusion [10]. In order to do this in a minimum of time, a standardised inter-professional and interdisciplinary algorithm is of utmost importance [11]. Such an algorithm should include an evidence-based cascade of diagnostic procedures and involve only those medical specialties that are definitely required for the evaluation and management of disorders of consciousness. This is a matter of particular interest especially in countries where a board-certification for in-hospital emergency physicians does not (yet) exist and where initial management of comatose patients in emergency departments (EDs) is frequently carried out by anaesthesiologists, internal specialists or surgeons, even in cases of focal CNS-pathologies such as intracerebral haemorrhage, meningo-encephalitis or basilar artery occlusion.

In Germany, on-site management of patients with disorders of consciousness is carried out by paramedics and trained emergency physicians. In order to assure a smooth and rapid workflow, out-of-hospital teams announce their patients in advance to the ED and state the leading emergency symptom. Based on this information, ED staff may trigger an appropriate in-house alarm before the estimated time of the patient’s arrival. This essentially involves a simultaneous alert call to a standardised group of recipients. The aim of such an alarm routine is to minimise delays and to ensure a direct contact between out-of-hospital teams and in-hospital physicians. Our university hospital campus (Charité-Universitätsmedizin Berlin, Campus Virchow-Klinikum) cares for approximately 100.000 emergency patients per year [12]. However, this number does not include patients who are comatose due to an obvious reason, such as an evident cardio-respiratory arrest, and thus admitted directly to specialised internal medicine intensive care units (ICUs). In past years, adult patients presenting to our ED with non-traumatic disorders of consciousness of unknown origin used to be treated along a well-established shock trauma centre protocol (level I trauma centre) that requires a team of trauma surgeons, anaesthesiologists and radiologists as well as a neurosurgeon in the first line. If, after initial assessment based on Advanced Trauma Life Support (ATLS^®^) [13], there had not been any clinical evidence for a traumatic reason explaining the patient’s state, the patient would then be moved on to CT scanning. Only after CT scans had excluded signs of traumatic brain injury (TBI) or intracranial haemorrhage, other medical specialties would have been involved. However, since secondary (e.g. metabolic, hypoxic etc.) causes of brain dysfunction account for a high percentage of underlying pathologies that is similar to the percentage of patients suffering from focal lesions of the CNS [7], this practice frequently caused delays in diagnosis and treatment of both medical and non-haemorrhagic neurological conditions.

To improve the management of adult non-traumatic patients presenting with a disorder of consciousness or coma in our hospital, an interdisciplinary expert panel developed a new in-house standard operating procedure (SOP) that includes a new in-house triage process, a new alert call, a new composition of the clinical response team and a new management algorithm (altogether termed “coma alarm”). The main goal of this feasibility study was to evaluate the success of the implementation of the SOP into a working ED environment including the sensitivity of the in-house triage process during a period of 24 months following its introduction in May 2013.

## Methods

### Procedure

**Fig. 1:**
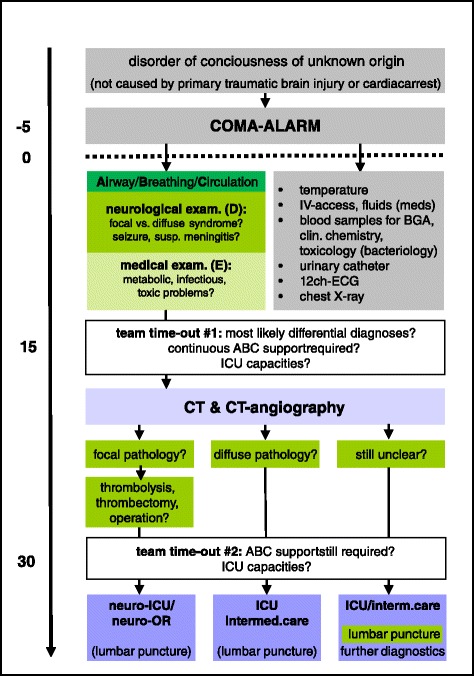
New interdisciplinary and inter-professional SOP for coma management in our ED; shown in relation to the target time scale (*left*); grey areas mark tasks to be performed by nursing staff, green areas highlight the physicians’ duties and responsibilities

We designed a new management protocol termed “coma alarm” for emergency patients presenting to the hospital with a disorder of consciousness without an obvious origin such as cardiac arrest or TBI. This includes patients with coma proper, sopor or deep somnolence (disorders of alertness or “quantitative” consciousness), i.e. all patients who are not fully awake. In order to identify patients eligible for the new protocol, two simple criteria are used. Based on the information received from out-of-hospital emergency teams, the head nurse checks a) whether the patient is reported to be fully awake when contacted by paramedics or emergency physicians and b) whether there is evidence of primary TBI. If both questions are negated, a non-traumatic disorder of consciousness of unknown origin is considered and staff are instructed to trigger the newly implemented coma alarm. The corresponding standardised interdisciplinary and inter-professional management of patients with a reduced level of consciousness (primarily comatose patients) is shown in Fig. 1. In contrast to an already well-established trauma response team, the coma alarm team consists of a neurologist as the team leader and an internal specialist accompanied by an anaesthesiology support team for airway (ABC) management. On the one hand, this arrangement accounts for the fact that patients with a disorder of consciousness of unknown origin may require highly urgent treatment of primary focal CNS-disease such as thrombolysis, mechanical thrombectomy, antibiotics or neurosurgery. On the other hand, said patients may suffer from severe medical conditions affecting the CNS secondarily (e.g. insufficiency of circulation or respiration, metabolic disturbance, sepsis etc.) [7] which require immediate diagnosis and management by internal specialists. A further difference is the participation of only one trauma surgeon required to differentiate primary TBI from secondary fall-related trauma upon loss of consciousness – a frequent question during initial management. Furthermore, the neurosurgeon on call receives a notification of the alarm but is not part of the initial response team. There are no differences in the number of nursing staff required as compared to the trauma call (Table 1). In both in-house alarms, one CT scanner in the radiology department is blocked so that a scan can be carried out without delay. The out-of-hospital emergency team leader presents the patient to the in-house team headed by the neurologist on call. While the anaesthesiology team carries out ABC management immediately, the neurologist is responsible for history taking (including as much information as possibly available from third parties) and neurological examination. At the same time, the internal specialist conducts the medical examination and evaluates chest X-ray and 12-channel-ECG as soon as ED radiographers and nursing staff carry them out. Besides, the nursing team draws blood samples for a standardised set of laboratory tests including blood gas analysis, basic toxicology and bacteriology (if suspected) and performs urinary catheterisation. Special emphasis is put on team time-outs. In surgery, team time-outs are meant to be the last security check immediately before a procedure begins to ensure that the correct patient undergoes the correct procedure on the correct side [14]. We modified this tool and use the first team time-out after completing the clinical evaluation of the patient (approximately 15 min after arrival) to update the whole team on all currently available information and the most likely differential diagnosis. Furthermore, both team time-outs are necessary to evaluate whether all personnel alarmed and prompted to the emergency room are still required. For example, anaesthesiology support might no longer be needed if a patient turns out to have continuously stable vital signs or to be awakening from a temporary loss of consciousness. Another important issue is to check for available intensive care capacities in the early stages of management to avoid delays. CT-scanning includes cranial CT plus supra-aortic CT-angiography and can be extended if needed (e.g. thorax for suspected pulmonary embolism). If CT-scans reveal a focal CNS pathology, immediate treatment is initiated (e.g. systemic thrombolysis or endovascular thrombectomy) or the patient is presented to the neurosurgeon on call. The nature of the underlying pathology in combination with the patient’s clinical condition determine whether the patient has to be admitted to an intensive care unit (ICU) or will be kept in intermediate care until further clarification. If medical history, physical examination, CT scans and primary laboratory results do not provide a compelling cause of the disorder of consciousness, a lumbar puncture has to be performed as soon as possible.

**Table 1:** Number of staff required for the in-house initial response teams; new coma alarm compared to well-established multiple trauma alarm

Coma alarm	Multiple trauma alarm
Neurologist	
Internal specialist	
Anaesthesiologist	Anaesthesiologist
Trauma surgeon	3 Trauma surgeons
Neurosurgeon (notification only)	Neurosurgeon
	Radiologist
2 Anaesthesiology nurses	2 Anaesthesiology nurses
3 ED nurses	3 ED nurses
2 Radiographers	2 Radiographers

### Data analysis

An ethics vote for the analysis of routinely acquired clinical data was obtained from the Ethics Commission of the Charité-Universitätsmedizin Berlin (“Emergency Processes in Clinical Structures”, EA1/172/14). All coma alarms between May 2013 and April 2015 were identified retrospectively in our hospital’s electronic patient database. We analysed all available documentation of each respective patient and determined a) whether the patient was in a state of reduced consciousness when contacted by paramedics or emergency physicians on-site, b) whether the patient had a disorder of consciousness on admission to the ED as determined by the team-leading neurologist and c) if there was any evidence of multiple trauma including primary TBI. Patients who had either been documented to be fully awake at all times (no persistent or transient loss of consciousness) or who presented with evidence of multiple trauma were defined as overtriaged or “false alarms”. All emergency referrals with a diagnosis already known were classified likewise as there was no need for further diagnostic procedures. Besides, we analysed all other emergency patients seen by a neurologist in the ED between May 2013 and April 2015 and applied the criteria mentioned above in order to identify undertriaged patients or “misses”.

## Results

**Fig. 2:**
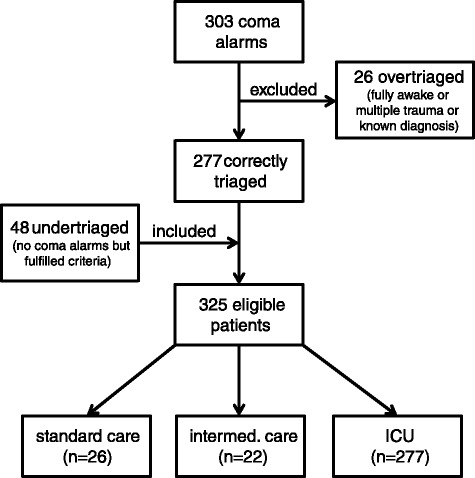
Identification of “hits”, “misses” and “false alarms” over the course of 24 months following the introduction of the new coma alarm by retrospective analysis of all available clinical documentation; bottom line abstracts subsequent in-house treatment; absolute numbers are given

Between May 2013 and April 2015, we registered 303 coma alarms in our ED (12.6 per month; see Fig. 2). Among these, 26 (8.6 %) had to be classified as “false alarms” retrospectively and excluded because patients had either been fully awake at all times (18 cases) or had been stabilised and did not require any further diagnostic procedures (7 cases) or had suffered multiple injuries including primary TBI resulting from an accident (1 case). In addition to 277 patients who were correctly triaged for the coma alarm emergency management routine by the two-step in-house triage process explained above (“hits”), we identified 48 undertriaged patients from the same period of time who fulfilled all criteria for the coma alarm and its dedicated emergency pathway but had not received it (“misses”). Nine patients out of these had not received any alarm-triggered standardised management and four had been classified as stroke alarm patients due to a focal neurological deficit suspected by paramedics or out-of-hospital emergency doctors. In both groups, there had been no information about the disorder of consciousness when the patients had been announced to the ED before arrival. A further 18 out of the 48 undertriaged patients had been announced as trauma patients due to a suspected accident as a cause of the loss of consciousness. They were treated along the well-established trauma management routines triggered by the corresponding alarm although an accident or multiple trauma could never be confirmed in these cases. Finally, 17 patients presenting with a loss of consciousness as the leading symptom were announced by the emergency services for an immediate referral to the neurosurgeon on call based on the assumption of an intracranial haemorrhage although a definite diagnosis had not yet been established.

Thus, we included a total of 325 patients presenting with non-traumatic disorders of consciousness of unknown origin as the leading symptom (182 male, median age: 66 years, median GCS: 6) that were or should have been treated along the newly established coma alarm management routine (277 “hits” + 48 “misses” = 325 eligible patients). Patient characteristics including the final diagnosis are given in Table 2. 277 patients (85.2 %) had to be admitted to ICUs. Among these, 111 (40.1 % of all ICU patients) had to be treated on specialised neuro-ICUs for primary, CNS-related causes of disorders of consciousness. 26 eligible patients (8.0 %) were admitted to standard care wards and another 22 (6.8 %) were treated in intermediate care units attached to the ED. Of all 325 eligible patients reported here, 75 (23.1 %) had a lethal outcome in the immediate course following admission to our hospital (median survival time 48 h, range <1 h to 29.3 days).

**Table 2:** Characteristics and outcomes for patients evaluated in this study (*n* = 325)

Characteristic	Median	Range
Age (years)	66	15–98
GCS	6	3–12
	*n*	%
Male	182	56,0
Diagnosis		
Intracranial haemorrhage	73	22,5
Ischaemic stroke	31	9,5
Epilepsy	66	20,3
Other neurologic	19	5,8
Cardiovascular	19	5,8
Respiratory	18	5,5
Metabolic	17	5,2
Septic	9	2,8
Intoxication	54	16,6
Other	19	5,8
With abnormality on CT scan	110	33,8
Admitted to ICU	277	85,2
Hospital mortality	75	23,1

Among the 277 patients correctly triaged for the coma alarm management routine (“hits”), 266 patients received CT scans of head and, if necessary, thorax and abdomen after an average door-to-CT time of 20 min (median; interquartile range 16–27 min) covering handover of the patient, stabilization of vital parameters, clinical examination and nursing tasks in the emergency room (see Fig. 1). 11 patients were not CT-scanned but received emergency MR scans instead (2 cases) or deceased in the resuscitation room (1 case) or presented with unmistakable evidence of either asphyxiation or intoxication and were not scanned in time due to psychomotor agitation (8 cases).

**Fig. 3:**
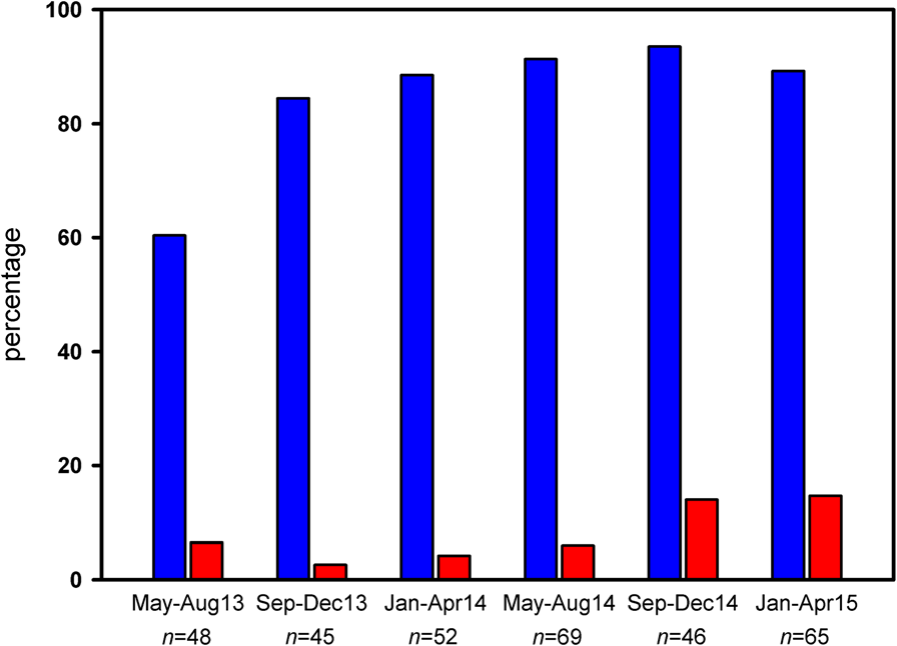
Sensitivity of the the newly established in-house triage process (*blue bars*) and false alarm rates (*red bars*) over the course of 24 months following the introduction of the SOP; time scale is binned into periods of four months each; absolute number of eligible patients in each time period is given below the groups of bars

Figure 3 shows the time-related changes in sensitivity (proportion of eligible patients correctly triaged for the alarm-triggered emergency management before their arrival in the hospital) and false alarm rates (proportion of overtriaged patients among all coma alarms) of our newly established in-house triage process preceding the specialised emergency management. The bar chart can be read as a learning curve over the course of 24 months. After the first four months, sensitivity increased from 60 to 84 %. In subsequent periods, sensitivity fluctuated between 89 and 94 %. Thus, undertriage could be kept low at a maximum rate of 11 % in any four-month observation period after an initial learning period of 8 months. 39 out of 48 undertriaged patients were treated along different management routines other than the newly established coma alarm which is equal to only 9 patients out of 325 eligible patients receiving no alarm-triggered management routine at all. Overtriage never exceeded 15 % during the whole observation period.

## Discussion

For patients with multiple trauma, clinical pathways and structured education concepts such as ATLS^®^ are well established in most EDs. Implementation of clinical algorithms is feasible [15, 16], shortens the time to diagnosis and treatment, and has been shown to significantly improve patient outcome [17, 18]. Similarly, in common medical and neurological emergencies such as myocardial infarction [19] and stroke [20], clinical pathways and implemented algorithms accelerate clinical workflow and improve outcome [21, 22]. Surprisingly, at least to our knowledge, comparable pathways for disorders of consciousness have not yet been universally implemented.

Here, we established a new ED management routine specifically designed for patients with the leading symptom of a non-traumatic disorder of consciousness of unknown origin. The regular admission of these patients to our ED justified the need for a specialised management that particularly focuses on rapid identification and treatment of possibly life-threatening underlying disorders. The new SOP thus includes an in-house alarm call triggered by the information on the patient’s state of consciousness given by out-of-hospital emergency services. By adhering to the new simple in-house triage criteria eligible patients could be properly identified in up to 94 % of the cases after an initial learning period.

We deliberately chose not to set a specific GCS criterion to identify eligible patients for the protocol because of the GCS’s unsatisfactory inter-rater reliability [23] and its insufficiency in correctly assessing the level of consciousness in patients with focal and/or lateralized neurological symptoms. A neurologist, an internal specialist, an anaesthesiology team, three ED nurses, two radiographers and a backup trauma surgeon are prompted to the ED. These staff and equipment requirements are comparable to resources required for management of multiple trauma patients in the ED [24]. We decided on the neurologist to lead the emergency team, coordinate the diagnostic steps, collect the patient’s past medical history, if available, and define the most likely diagnosis. This is done in close collaboration with the other members of the team, given that non-traumatic disorders of consciousness can be caused by either primary brain disease or secondary diffuse neuronal dysfunction [1]. In multiple trauma management, it is still a matter of debate whether there should be a single team leader or a team-guided approach [25]. However, the need for structured guidance and supervision of the process is beyond any dispute [26]. Although we did not systematically evaluate different team structures, we deem appropriate our hybrid approach that includes team-guidance by a neurologist and obligatory interdisciplinary team-time-outs for a highly interdisciplinary emergency like coma and other disorders of consciousness of unknown origin.

One of the main advantages of our protocol is gathering all required personnel on site before the patient arrives in order to ensure the fastest possible proceedings in time-sensitive cases. However, the protocol was deliberately designed to give the opportunity to reduce the level of resource allocation whenever incoming diagnostic results justifies to do so (e.g. 26 overtriaged patients were excluded from the cohort of eligible patients; CSF testing was not performed if preceding investigations provided positive and unequivocal results; etc.). Thus, team-time-outs play an important role not only for interdisciplinary consultation but they also allow for the level of resource allocation to be adjusted to any required level.

Given the high percentage of patients requiring intensive care management after admission, there is a need to use available resources as purposefully as possible. Furthermore, the high portion of life-threatening causes of coma and other disorders of consciousness of non-traumatic origin is reflected in the scope of diagnoses in combination with the remarkable percentage of patients who deceased in the immediate course following presentation to ED (Table 2). This is the strongest argument for a multi-disciplinary approach with an emphasis on primarily neurological and medical first-line assessment as opposed to a primary surgical management which is in fact mostly required to treat collateral problems (such as superficial wounds) without impact on the underlying pathologies in this cohort of patients.

Over 85 % of all eligible patients required intensive care treatment, over 40 % of which needed specialised neuro-intensive care or neurosurgical treatment following admission due to primary disorders of the CNS. A similar portion was treated in ICUs specialised in internal medicine. This is another argument for assigning the initial diagnostic workup to neurologists and internal specialists rather than to surgeons, with anaesthesiologists focusing on life support. Initial management does usually not require the immediate presence of a neurosurgeon, as initial neurological and neurosurgical evaluation of comatose patients do not differ. Nevertheless, whenever required (e.g. CT reveals subdural haematoma), our SOP allows for prompting the already pre-alerted neurosurgeon on call to the ED as quickly as possible.

The absolute number of eligible patients included in this study showed an upward trend over time, possibly indicating that establishing the SOP may have met a certain demand in our catchment area for a specialised emergency management for patients presenting with a disorder of consciousness without an obvious origin. Although the information provided by out-of-hospital emergency teams frequently was fragmentary, there was a surprisingly high sensitivity of the in-house triage process required to identify patients eligible for the SOP after the first four months. Hence it appears that the obligatory two questions to be asked by the head nurse communicating with out-of-hospital response teams may be sufficient to correctly identify the patients that our SOP is intended for. Overtriage was only 8.6 % and was tolerated throughout as “false alarms” may indeed result in a strain of hospital resources but do not put patients to any risk. Furthermore, our rate is lower than in other reports of newly implemented alarm routines, e.g. in trauma management [27]. After an initial learning period of 8 months, undertriage could be kept at an overall low rate of 9.5 %. The aim is to reduce this figure even further by continuous training of all personnel involved. We determined each respective patient’s door-to-CT time as a reliable marker of time efficiency of our newly established management routine. By a median time of 20 min we could achieve a shorter time than reported in other routines for multiple trauma [17, 28] or stroke [20]. However, a complete evaluation of this marker is limited since comparable data from eligible patients was not prospectively collected before the implementation of our new SOP.

## Conclusions

The successful implementation of an alarm-triggered management routine specifically designed for patients presenting with disorders of consciousness of unknown origin provides us with a powerful tool to optimise diagnostic work-up and treatment. Furthermore, the alarm-tagged cases will allow for systematic investigation of this fundamental emergency symptom in further detail. After proof of feasibility in this study, subsequent interventional studies based on a growing registry of clinical data will aim at evaluating the effects on outcomes, scrutinising the validity and priority of different diagnostic tools and ultimately establishing an evidence-based SOP “from street to ICU” for disorders of consciousness of unknown origin which is currently lacking in emergency medicine.
